# Handgrip Strength and Cognitive Recovery in Older Stroke Survivors: A Prospective Study

**DOI:** 10.3390/medicina60101697

**Published:** 2024-10-16

**Authors:** Young-Ah Choi

**Affiliations:** Department of Rehabilitation Medicine, Incheon St. Mary’s Hospital, College of Medicine, The Catholic University of Korea, Seoul 21431, Republic of Korea; simple16@catholic.ac.kr; Tel.: +82-32-280-6601

**Keywords:** cognitive dysfunction, handgrip strength, neuropsychological tests, rehabilitation, stroke

## Abstract

*Background and Objectives*: Handgrip strength (HGS) is an important indicator of overall physical capability and is linked to various health outcomes in older adults. Despite extensive research on the relationship between HGS and cognitive decline, longitudinal studies on poststroke cognitive changes in relation to HGS are scarce. This study aimed to observe whether HGS at discharge from acute stroke rehabilitation affects cognitive function 6–12 months after stroke onset and to compare cognitive outcomes between patients with normal and low HGS at discharge. *Materials and Methods*: This prospective cohort study was conducted in the Department of Rehabilitation Medicine at a tertiary care hospital. In agreement with the Asian Working Group for Sarcopenia 2019 criteria, low muscle strength was defined as an HGS of less than 28 kg for men and less than 18 kg for women, and participants were categorized into normal and low HGS groups. Neuropsychological evaluations were conducted before discharge (approximately one month after stroke onset) and between 6 and 12 months after stroke onset. *Results*: The low HGS group was older and had lower Montreal Cognitive Assessment scores. However, after adjusting for covariates, the linear mixed model analysis showed no significant differences between the groups in global cognition or specific cognitive domains, except for psychomotor speed during the subacute phase. Notable improvements in language ability were observed in both groups over time, while significant improvements in executive function were observed exclusively in the low HGS group. *Conclusions*: This longitudinal study is the first to explore the relationship between HGS and changes in cognitive function in older adults with stroke, providing insights into physical strength and cognitive recovery during stroke rehabilitation.

## 1. Introduction

Stroke is a major disease with sudden onset and can cause persistent disability. Widely recognized as a critical health concern, stroke affects older adults as global aging progresses [1,2]. Although survival rates and stroke severity have improved owing to advances in acute stroke treatment [3], poststroke cognitive impairment remains prevalent and often results in poor outcomes [4]. For example, if residual cognitive dysfunction remains 6 months after a stroke, patients experience poorer quality of life, reduced independence, increased informal care needs, and increased depressive symptoms 5 years after the stroke [5]. Increasing age is one of the risk factors for cognitive impairment after stroke [6], and cognitive decline following stroke is more frequently observed in the older adult population. Previous studies using registry data have reported a strong association between age and the prevalence of cognitive impairment among stroke survivors, noting a progressive increase in this prevalence among individuals aged 65–85 years from the 5th year after the stroke [7]. Additionally, a recent cross-sectional study in China focusing solely on the older adult population found a prevalence rate of approximately 40%, highlighting the high prevalence of poststroke cognitive impairment among older stroke survivors [8].

Physical ability is as important as cognitive function in older adults. Among the various indicators used to measure physical function, handgrip strength (HGS) has recently gained wide recognition as a marker of muscle function and overall physical capability [9]. Low HGS is not only associated with musculoskeletal disorders but also with several non-communicable diseases, such as stroke. For example, a longitudinal study found that a lower HGS is linked to a higher risk of stroke, making HGS an independent predictor of stroke in middle-aged and older populations in Europe, America, and China [10]. Additionally, low HGS has been identified as a global risk factor for cardiovascular diseases, including stroke and mortality [11], and is also known to be an important factor for higher rates of nursing home placement or hospitalization in older adults [12]. Conversely, a high HGS at baseline is a protective factor against mobility limitations, decline in functional status, and mortality [13].

Beyond physical aspects, a low HGS is associated with psychological health problems, particularly cognitive impairment. Studies have shown that older adults in the highest HGS quartile have significantly lower likelihoods of cognitive decline—62% for men and 49% for women—compared to those in the lowest quartile [14]. Lower HGS has also been linked to a higher risk of developing mild cognitive impairment and a faster decline in various cognitive functions [15]. However, other studies have found no association between HGS and cognitive decline. For instance, long-term follow-ups of community-dwelling individuals found that HGS did not predict cognitive decline [16,17]. Despite some inconsistencies regarding the association between HGS and cognitive outcomes in the general older population, numerous studies have reported on this relationship, utilizing both cross-sectional and longitudinal designs. Cognitive decline is more prevalent among patients with stroke and is closely related to poor outcomes. While some studies have reported that HGS is associated with the prognosis of patients with stroke, most of these studies are cross-sectional, and research specifically examining the relationship between HGS and poststroke cognitive function in stroke survivors is notably lacking. Given the highly variable and complex trajectory of cognitive recovery in stroke survivors, longitudinal studies that use biomarkers like HGS to predict and monitor cognitive changes over time are needed.

Therefore, this study aimed to prospectively assess whether HGS at the time of discharge from an acute stroke rehabilitation ward influences cognitive function 6–12 months after stroke onset. Because previous research suggests that most of the cognitive recovery occurs within the first 6 months following a stroke [18], it was deemed appropriate to conduct follow-up assessments at least 6 months after the stroke—a period during which cognitive changes tend to stabilize. The hypothesis was that a lower HGS at discharge would be associated with poorer cognitive outcomes during the follow-up period. Furthermore, given reports indicating that different cognitive domains may recover at varying rates after a stroke [18,19], it was posited that the recovery of specific cognitive functions might also differ depending on the HGS at discharge. Therefore, the objective was to examine differences in overall cognitive function and specific cognitive domains between patients with normal and low HGS at discharge. By categorizing patients into groups with normal or low HGS at discharge following acute rehabilitation, this study aimed to explore not only the trajectory of overall cognitive recovery but also to test the hypothesis that the degree of recovery in specific cognitive functions varies according to the HGS group.

## 2. Materials and Methods

### 2.1. Participants and Study Setting

This prospective observational study was conducted in the Department of Rehabilitation Medicine at a tertiary care hospital between August 2021 and February 2023. All eligible patients with stroke were diagnosed at the Department of Neurology or Neurosurgery and were transferred to the neurorehabilitation unit once they were medically stable. The inclusion criteria were (1) age 65 years or older, (2) first-ever stroke with evidence of cerebral infarction or intracerebral hemorrhage on magnetic resonance imaging or computed tomography, and (3) patients were able to complete the skeletal muscle mass index (SMI) and HGS measurements. The exclusion criteria included prestroke functional limitations as indicated by a modified Rankin Scale (mRS) score of 3 or higher and active comorbid diseases. Furthermore, patients with severe disturbances of consciousness or communication disorders, such as global aphasia, acute delirium, dementia, or major psychiatric disorders, diagnosed by a physician prior to stroke onset were excluded.

### 2.2. Sample Size Calculation

The required sample size was calculated using PASS 12.0 software with a two-sided two-sample equal-variance *t*-test [20,21,22,23]. Drawing on data from a previous study [24], which showed a mean difference of 3.3 points in Mini-Mental State Examination (MMSE) scores between normal and sarcopenic groups, a more conservative standard deviation of 4.6 was assumed. Thus, a minimum of 80 patients was required to achieve sufficient statistical power (α = 0.05, β = 0.1). To account for a potential dropout rate of 25%, the sample size was adjusted to at least 108 participants.

### 2.3. Low HGS Group vs. Normal HGS Group

According to the Asian Working Group for Sarcopenia (AWGS) 2019, low muscle strength is defined as an HGS of less than 28 kg in men and less than 18 kg in women [25]. HGS was measured three times using a Jamar dynamometer (Model 081028950), and the highest value was recorded. The cutoff values for low SMI were set at less than 7.0 kg/m^2^ for men and less than 5.7 kg/m^2^ for women, as determined using a portable bioelectrical impedance analysis device (INBody S10, InBody Japan, Tokyo, Japan). To specifically assess the HGS effects, only patients with normal SMI values, as defined by the AWGS cutoff, were included in the analysis. Based on the HGS at the time of discharge, participants were categorized into low and normal HGS groups according to the AWGS 2019 cutoff criteria.

### 2.4. Data Collection

Sarcopenia was evaluated using HGS and SMI at the time of transfer to the neurorehabilitation unit, and a follow-up evaluation was performed before discharge after 4 weeks of stroke rehabilitation. Baseline patient characteristics included age, sex, body mass index, prestroke mRS score, comorbidity severity (age-adjusted Charlson comorbidity index), years of education, stroke type (ischemic vs. hemorrhagic), and neurological severity using the National Institute of Health Stroke Scale (NIHSS).

### 2.5. Neuropsychological Evaluation

Neuropsychological evaluations were conducted before discharge (approximately one month after stroke onset) and followed up between 6 and 12 months after stroke onset to assess late poststroke cognitive function. During the follow-up period, patients did not receive separate standardized cognitive interventions. Cognitive function was assessed using the Korean version of the Montreal Cognitive Assessment (MoCA). Patients with no more than 6 years of education received an additional point in their total MoCA score. MoCA scores range from 0 to 30, with higher scores indicating better cognitive function. The comprehensive Seoul Neuropsychological Screening Battery [26], which includes standardized and validated measures across various cognitive domains, was administered to all participants. For this study, assessments were selected that provide numerical outcomes: the Digit Symbol Coding (DSC) task to assess processing speed and working memory and the digit span tests (DST), both forward and backward, to evaluate attention. Language function was assessed using the Korean version of the Boston Naming Test (BNT), and visuospatial function was evaluated using the Rey–Osterrieth Complex Figure Test (RCFT):Copy task. Verbal memory was measured using the 20-min delayed recall (DR) of the Seoul Verbal Learning Test (SVLT), while visual memory was assessed using the DR of the RCFT. The Controlled Oral Word Association Test (COWAT) evaluated categorical and phonemic generative naming ability, a sensitive marker of frontal lobe function. Frontal or executive function, visual attention, and task switching were assessed using Parts A and B of the Trail Making Test (TMT).

### 2.6. Statistical Analysis

Continuous variables are presented as means and standard deviation (SD). Categorical variables are presented as counts and percentages. Continuous variables were analyzed using either the independent t-test for normally distributed data or the Wilcoxon rank-sum test for non-normally distributed data. Categorical variables were compared using Pearson’s chi-square test or Fisher’s exact test, as appropriate. Initial comparisons were made using raw scores adjusted for age, sex, and years of education to compare the differences in domain-specific cognitive function between the two groups at discharge and follow-up. Subsequently, generalized linear mixed models were used to evaluate differences between the groups according to HGS for global cognitive function testing using the MoCA and all domain-specific cognitive function test scores between discharge and 6–12 months follow-up with group, time, and their interaction. This analysis adjusted the raw scores of each cognitive domain assessment for NIHSS and discharge MoCA scores, as well as age, sex, and years of education. The model was adjusted for each patient’s follow-up duration to account for variability in follow-up intervals. After statistically confirming that the follow-up duration did not influence the outcomes, this variable was excluded from the final analysis. Post hoc tests were conducted using the Bonferroni method. After generalized linear mixed analysis, the cognitive test results were expressed as adjusted least square means after controlling for group, time, and other covariates (age, sex, education, NIHSS score, and baseline MoCA score). To confirm an adequate sample size and ensure sufficient statistical power, a post hoc power analysis was conducted using two-sample t-tests with unequal variance. The significance level (alpha) was set at 0.050. These calculations of post hoc power analysis were based on methodologies outlined in Julious (2023), Chow et al. (2017), Machin et al. (2011), and Zar (1999) [20,21,22,23]. *p* < 0.05 was considered statistically significant. Statistical analyses were performed using SAS software (version 9.4, SAS Institute, Cary, NC, USA) and R (version 4.3.1).

## 3. Results

### 3.1. Patient Characteristics According to HGS

Of the initial 100 patients who were followed up, 50 were excluded for the following reasons: twenty-six patients became bedridden to the extent that they could not use a wheelchair, six refused follow-up evaluations, five were unable to attend due to COVID-19 quarantine restrictions, five passed away, six relocated to a distant region, and two experienced a recurrent stroke. Finally, 50 patients completed the 6–12-month follow-up period and were included in the analysis. Table 1 summarizes the baseline characteristics of the participants. The mean age of the older adults with stroke was 74.96 ± 6.78 years, and 18 (36%) patients were men. The baseline patient characteristics according to HGS at discharge are shown in Table 2. Compared with the normal HGS group, the low HGS group was older (76.76 ± 6.11 vs. 72.48 ± 7.01, *p* = 0.03). Furthermore, patients with low HGS had significantly lower MoCA scores at discharge (14.00 ± 7.02 vs. 19.14 ± 6.81, *p* = 0.01) compared to those with normal HGS. The follow-up was conducted 8.93 ± 2.31 and 9.21 ± 2.52 months after the stroke in the low HGS and normal HGS groups, respectively, with no significant difference in follow-up timing between the two groups (*p* = 0.72). At the follow-up assessment, the MoCA scores remained lower in the low HGS group than in the normal HGS group (12 ± 12 vs. 22 ± 8, *p* = 0.002).

### 3.2. Generalized Linear Mixed Model

Table 3 presents the results of the generalized linear mixed model used to analyze the differences in global cognitive function and specific cognitive domains between the low and normal HGS groups over time (discharge vs. follow-up).

**Figure 1:**
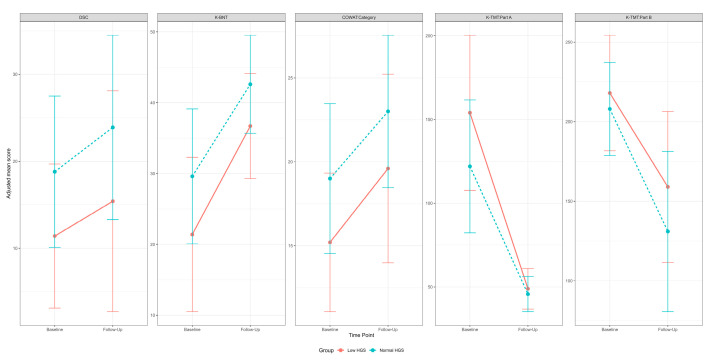
Adjusted mean (standard deviation) of raw scores for each group in the DSC test, K-BNT, COWAT-Category, and Parts A and B of the K-TMT, adjusted for age, sex, and years of education.

The generalized linear mixed model did not identify any significant interactions between group and time across all subtests, nor were there significant differences in MoCA scores between groups or across time points. However, significant main effects for either group or time were observed in specific cognitive domain tests, including the DSC, BNT, COWAT-Category, K-TMT Part A, and K-TMT Part B. In the DSC test, a significant group effect was observed, with the normal HGS group scoring significantly higher than the low HGS group at discharge, indicating that psychomotor speed was significantly lower in the low HGS group (*p* = 0.01, post hoc *p* = 0.03). The BNT showed a significant effect of time (*p* < 0.0001), with both groups demonstrating significant improvements in confrontational naming ability from discharge to follow-up at 6–12 months after the stroke (low HGS: *p* < 0.0001, normal HGS: *p* = 0.002). The COWAT-Category, K-TMT Part A, and K-TMT Part B tests all indicated significant time effects (*p* = 0.01, *p* < 0.001, and *p* < 0.001, respectively), with significant improvements in executive function assessment scores observed exclusively in the low HGS group from discharge to follow-up (COWAT-Category: *p* = 0.02, K-TMT Part A: *p* < 0.001, K-TMT Part B: *p* = 0.02). Figure 1 illustrates the adjusted mean and standard deviation scores, adjusted for age, sex, and years of education, to visualize the trends across different groups and time points. No significant group or time effects were detected for global cognitive function or other domain-specific cognitive functions. Even when significant main effects were noted, post hoc analyses did not confirm them.

### 3.3. Post Hoc Power Analysis

The post hoc power analysis indicated that with group sample sizes of 29 and 21, a power of 96.20% was achieved to detect a significant difference in means, assuming a population mean difference in MMSE scores of −6.7 (low HGS group = 17.9 vs. normal HGS group = 24.6) and standard deviations of 8.3 for the low HGS group and 3.9 for the normal HGS group. As the prior study used for the sample size calculation assessed cognitive function using the MMSE, MMSE scores were also included in this post hoc power analysis [24]. Furthermore, multiple simulations have demonstrated that with at least 12 participants, variance tends to stabilize, which supports the reliability of the statistical power achieved in the current study [27].

## 4. Discussion

This study demonstrated that, after adjusting for confounding factors using a generalized linear mixed model, global cognitive function did not significantly differ between the low and normal HGS groups following acute stroke rehabilitation. However, differences were evident in specific cognitive domains, particularly in psychomotor speed during the subacute phase. Furthermore, grip strength did not appear to have a significant impact on changes in cognitive function up to 6–12 months after stroke onset. Notably, both groups exhibited improvements in language abilities over time, with the low HGS group showing exclusive improvements in executive function.

The low HGS group was older and had lower MoCA scores. HGS serves as an indicator of sarcopenia, encompassing age-related decline in muscle mass, strength, and function. A cohort study of individuals aged 70–95 years demonstrated that, on average, HGS declined progressively with age, with a 46.3% decrease in women and a 44.7% decrease in men [16]. Most epidemiological studies focusing on the association between HGS and the risk of cognitive decline have been conducted in community-dwelling older populations, and the results have been controversial. As this study is the first to longitudinally track cognitive function in older patients with first-ever stroke from the subacute to the chronic phase and to evaluate the association between HGS and cognitive decline, it is challenging to make direct comparisons with previous research that focused on the general older population. Although MoCA scores were initially lower in the low HGS group during both the subacute and chronic phases, no significant differences were observed after adjusting for stroke severity and baseline cognition, as well as age, sex, and education, in older adults who had recently experienced a stroke. This suggests that the substantial impact of stroke on cognitive function likely overshadowed any differences related to HGS.

The absence of a significant interaction effect between group and time in the generalized linear mixed model suggests that cognitive function trends over time were similar across groups, indicating that HGS measured during the subacute stage does not influence cognitive changes up to 6–12 months after the stroke. While some previous studies have recommended using HGS to monitor cognitive changes over time [28], these recommendations may not be applicable to older adults in the subacute-to-early-chronic poststroke phase. A study examining the temporal relationship between cognitive performance and HGS in a very old population found that baseline cognitive performance predicted a subsequent decline in HGS [16]. However, consistent with the findings of the current study, baseline HGS was not significantly associated with subsequent cognitive decline. Further research is needed to explore the temporal relationship between HGS and cognitive function in older adults who have experienced a stroke.

The DST is a highly sensitive measure of cognitive function that is particularly effective in detecting subtle changes and variations in general processing speed and higher-level cognitive function, outperforming the MMSE [29]. The DST is particularly valued for its brevity, sensitivity to cognitive changes, and minimal influence of educational and cultural backgrounds [30]. Previous research has demonstrated significant associations between HGS and DST performance. For example, a longitudinal study in community-dwelling older adults in Japan found that lower HGS was significantly associated with lower DST scores [31], whereas a study of female cancer survivors in the United States showed that higher HGS correlated with better DST performance, suggesting a positive relationship between HGS and cognitive function [32]. Similarly, another study found that greater quadriceps strength was linked to better psychomotor performance, although the exact biological mechanisms behind these associations remain unclear. Possible explanations include vascular dysfunction, inflammation, social engagement, and metabolic changes, but these theories require further investigation [33]. In the current cohort, differences between HGS groups persisted even after adjusting for stroke severity during the subacute phase, suggesting that those in the low HGS group may have had reduced psychomotor speed before the stroke. However, these differences were no longer significant during the follow-up period, likely due to the natural course of cognitive recovery that reduced the initial disparities between the groups.

The BNT is among the most widely used naming assessments in contemporary neuropsychological evaluations [34]. A study involving patients with acquired language impairment due to acute neurological damage found that older participants, specifically those aged >55 years, showed significant annual improvements in BNT scores. Researchers have inferred that if these individuals also experience age-related cognitive decline, their actual overall improvement is slightly higher. This suggests that the impact of aging is relatively minor compared to long-term recovery from acquired language impairment [35]. In line with a previous study, the senior cohort of the current study also showed improvement in confrontational naming ability over time, up to 6–12 months after the stroke in both groups, regardless of HGS.

A noteworthy finding was that significant improvements in executive function assessment scores (DSC, COWAT-Category, K-TMT Part A, and K-TMT Part B) were exclusively observed in the low HGS group. Research on cognitive impairment following stroke has revealed a considerable prevalence of executive function deficits but has also shown significant recovery in this area compared to other cognitive domains. For instance, 15–50% of patients with early poststroke executive function impairments exhibit significant recovery within the following year [19,36]. A recent meta-analysis found that while there was an overall cognitive improvement in the first year after the stroke, executive function often showed sustained improvement, in contrast to declines in other cognitive areas [37]. Additionally, Turunen et al. reported that 60% of patients improved significantly at 6 months after the stroke, with minimal further change at 2 years [18]. In the cohort of the current study, even after adjusting for confounding factors, significant improvements in executive function were observed only in the low HGS group, suggesting that this may be inherently linked to their characteristics. Low HGS and executive function decline may share common mechanisms, as both involve brain regions related to motor control and cognitive functions. Structural changes, such as reduced brain volume and white matter alterations, might contribute to the link between poor physical condition and cognitive decline [38,39,40,41]. Further research is needed to clarify the mechanisms behind the observed improvement in executive function in the low HGS group. One possible explanation is that individuals with low HGS may engage compensatory neural mechanisms to offset deficits. Additionally, physical frailty may trigger targeted rehabilitation interventions that specifically enhance executive function [42], while psychosocial factors, such as increased social support or motivation during rehabilitation, might also play a role.

The strength of this study lies in its pioneering investigation of the relationship between HGS and changes in cognitive function in older adults with stroke, encompassing both general and domain-specific cognitive functions. However, this study has a few limitations. First, the relatively small sample size may have affected the generalizability of this study’s findings. A larger sample size would enhance statistical power to detect smaller differences and improve external validity. Second, only one follow-up between 6 and 12 months after stroke was included, which may have caused us to miss smaller or more gradual changes in cognitive function over time. Due to the COVID-19 pandemic during the study period, the follow-up assessments were limited to a window of 6–12 months. More frequent assessments would offer a clearer understanding of cognitive changes over time. Third, attrition bias caused by the loss of patients to follow-up might have occurred, which may affect the validity of the current results, particularly if those lost differed systematically from those who completed the study. To address this, post hoc analyses were conducted to determine whether significant differences existed between the characteristics of patients lost to follow-up and those who remained in the study. This analysis did not reveal any significant differences, suggesting that attrition may not have substantially influenced the current findings; however, some degree of bias cannot be entirely excluded. Fourth, Imaging data, such as stroke lesions and volumes, were not included in the analysis. While baseline cognitive function was adjusted for initial cognitive function using the baseline MoCA score in the generalized linear mixed model, imaging data can provide more precise adjustments for stroke lesions and volumes. Finally, the use of the AWGS 2019 criteria to define low and normal HGS may limit the generalizability of this study’s findings to non-Asian populations. Future studies should assess whether these findings are consistent across different demographic groups.

While HGS itself does not directly influence cognitive function changes from the subacute to the chronic phase after stroke, given the significant recovery observed in executive function in the low HGS group, further research is essential to elucidate the mechanisms behind the pronounced improvement in executive function observed in the low HGS group. This understanding could lead to the development of targeted therapies aimed at maximizing the recovery potential of executive functions in this fragile group.

## 5. Conclusions

This study is the first to explore the relationship between HGS and changes in cognitive function in older adults with stroke, tracking them longitudinally from the subacute to the chronic phase. The results showed that HGS during the subacute phase did not predict cognitive decline in the chronic phase (6–12 months after the stroke). Notably, psychomotor speed was lower in the low HGS group at discharge, but both groups showed improved confrontational naming ability over time, with significant executive function improvements observed exclusively in the low HGS group.

## Figures and Tables

**Table 1:** Baseline characteristics of the study participants.

	Total (N = 50)
Age (years)	74.96 (6.78)
Sex	
Male	18 (36.00%)
Female	32 (64.00%)
BMI (kg/m^2^)	23.81 (3.92)
mRS (points)	0.46 (1.13)
CCI (points)	
≤4	34 (68.00%)
5–6	14 (28.00%)
≥7	2 (4.00%)
Education (years)	7.74 (4.41)
Lesion	
Ischemic	39 (78.00%)
Hemorrhagic	11 (22.00%)
NIHSS (points)	4.82 (4.72)
MoCA at discharge (points)	16.16 (7.32)

Data are presented as mean (SD) or N (percentage). BMI = body mass index, CCI = Charlson comorbidity index, MoCA = Montreal Cognitive Assessment, mRS = modified Rankin Scale, NIHSS = National Institute of Health Stroke Scale.

**Table 2:** Comparative analysis of the two study groups based on handgrip strength.

	Low HGS (N = 29)	Normal HGS (N = 21)	*p*-Value
Age (years)	76.76 (6.11)	72.48 (7.01)	0.03 (T)
Sex			0.15 (C)
Male	8 (27.59%)	10 (47.62%)	
Female	21 (72.41%)	11 (52.38%)	
BMI (kg/m^2^)	24.15 (4.57)	23.34 (2.82)	0.45 (T)
mRS (points)	0.4 (0.8)	0.1 (0.4)	0.12 (W)
CCI (points)			0.49 (F)
≤4	18 (62.1%)	16 (76.2%)	
5–6	10 (34.5%)	4 (19%)	
≥7	1 (3.4%)	1 (4.8%)	
Education (years)	6.83 (4.36)	9.00 (4.25)	0.08 (W)
Lesion			0.32 (F)
Ischemic	21 (72.41%)	18 (85.71%)	
Hemorrhagic	8 (27.59%)	3 (14.29%)	
NIHSS (points)	5.38 (5.44)	4.05 (3.46)	0.71 (W)
MoCA at discharge (points)	14.00 (7.02)	19.14 (6.81)	0.01 (T)

Data are presented as mean (SD) or N (percentage). Between-group *p*-values: independent *t*-test (T) or Wilcoxon’s rank-sum test (W) for continuous data and chi-square test (C) or Fisher’s exact test (F) for categorical data. HGS = handgrip strength.

**Table 3:** Generalized linear mixed model.

		Low HGS	Normal HGS	*F*-Value	*p*-Value	Post Hoc *p*-Value
MoCA	Initial, LSM (SE)	15.38 (1.23)	17.08 (1.42)			
	1Y, LSM (SE)	14.73 (1.23)	18.08 (1.42)			
	Group			2.30	0.14	
	Time			0.03	0.86	
DSC	Initial, LSM (SE)	9.01 (2.81)	21.47 (3.25)			
	1Y, LSM (SE)	15.49 (2.81)	23.09 (3.25)			
	Group			7.48	0.01	0.03 (B)
						0.45 (F)
	Time			2.76	0.10	
DST:Forward	Initial, LSM (SE)	5.95 (0.30)	5.61 (0.35)			
	1Y, LSM (SE)	5.84 (0.30)	5.75 (0.35)			
	Group			0.27	0.60	
	Time			0.01	0.94	
DST:Backward	Initial, LSM (SE)	2.42 (0.25)	2.96 (0.29)			
	1Y, LSM (SE)	2.49 (0.25)	3.20 (0.29)			
	Group			3.60	0.06	
	Time			0.50	0.48	
K-BNT	Initial, LSM (SE)	21.93 (2.21)	27.54 (2.56)			
	1Y, LSM (SE)	38.38 (2.21)	39.06 (2.56)			
	Group			1.21	0.28	
	Time			50.93	<0.0001	<0.0001 (L)
						0.002 (N)
RCFT:Copy	Initial, LSM (SE)	9.82 (1.84)	14.05 (2.13)			
	1Y, LSM (SE)	12.67 (1.84)	13.09 (2.13)			
	Group			0.95	0.33	
	Time			0.34	0.56	
SVLT:DR	Initial, LSM (SE)	1.11 (0.35)	0.93 (0.41)			
	1Y, LSM (SE)	1.83 (0.35)	2.17 (0.41)			
	Group			0.03	0.86	
	Time			8.17	0.01	0.55 (L)
						0.11 (N)
RCFT:DR	Initial, LSM (SE)	2.99 (1.06)	3.18 (1.23)			
	1Y, LSM (SE)	2.67 (1.06)	4.99 (1.23)			
	Group			0.83	0.37	
	Time			0.63	0.43	
COWAT:Category	Initial, LSM (SE)	15.31 (1.55)	18.65 (1.79)			
	1Y, LSM (SE)	20.35 (1.55)	21.70 (1.79)			
	Group			1.31	0.26	
	Time			9.61	0.01	0.02 (L)
						0.66 (N)
COWAT:Letter	Initial, LSM (SE)	5.75 (1.57)	6.00 (1.81)			
	1Y, LSM (SE)	7.23 (1.57)	10.19 (1.81)			
	Group			0.60	0.44	
	Time			4.70	0.04	>0.99 (L)
						0.20 (N)
K-TMT:Part A	Initial, LSM (SE)	168.22 (16.38)	111.24 (18.98)			
	1Y, LSM (SE)	52.98 (16.38)	49.48 (18.98)			
	Group			2.45	0.12	
	Time			27.75	<0.001	<0.001 (L)
						0.10 (N)
K-TMT:Part B	Initial, LSM (SE)	234.20 (23.05)	203.25 (26.58)			
	1Y, LSM (SE)	166.44 (23.05)	138.25 (26.58)			
	Group			0.88	0.35	
	Time			14.16	<0.001	0.02 (L)
						0.10 (N)

Post hoc analysis using the Bonferroni method. The LSM and SE values are adjusted for group, time, and additional covariates, including age, sex, education level, NIHSS score, and baseline MoCA score. Note: Significant time × group interactions were not identified in any test; therefore, this interaction has been omitted from the table. Differences between groups: (B) baseline, (F) follow-up 6–12 months after the stroke; differences between time points: (L) low HGS, (N) normal HGS. COWAT = Controlled Oral Word Association Test, DR = Delayed Recall, DSC = Digit Symbol Coding, DST = Digit span tests, K-BNT = Korean version of the Boston Naming Test, K-TMT = Korean version of the Trail Making Test, LSM = least-square means, RCFT = Rey–Osterrieth Complex Figure Test, SE = standard error, SVLT = Seoul Verbal Learning Test.

## Data Availability

All data analyzed during this study are available from the corresponding author upon reasonable request due to privacy restrictions.

## References

[1] Michel J.P., Leonardi M., Martin M., Prina M. (2021). WHO’s report for the decade of healthy ageing 2021–30 sets the stage for globally comparable data on healthy ageing. Lancet Healthy Longev..

[2] Rajati F., Rajati M., Rasulehvandi R., Kazeminia M. (2023). Prevalence of stroke in the elderly: A systematic review and meta-analysis. Interdiscip. Neurosurg..

[3] Berkhemer O.A., Fransen P.S., Beumer D., van den Berg L.A., Lingsma H.F., Yoo A.J., Schonewille W.J., Vos J.A., Nederkoorn P.J., Wermer M.J. (2015). A randomized trial of intraarterial treatment for acute ischemic stroke. N. Engl. J. Med..

[4] Marsh E.B., Lawrence E., Hillis A.E., Chen K., Gottesman R.F., Llinas R.H. (2018). Pre-stroke employment results in better patient-reported outcomes after minor stroke: Short title: Functional outcomes after minor stroke. Clin. Neurol. Neurosurg..

[5] Rohde D., Gaynor E., Large M., Mellon L., Hall P., Brewer L., Bennett K., Williams D., Dolan E., Callaly E. (2019). The impact of cognitive impairment on poststroke outcomes: A 5-year follow-up. J. Geriatr. Psychiatry Neurol..

[6] Khedr E.M., Hamed S.A., El-Shereef H.K., Shawky O.A., Mohamed K.A., Awad E.M., Ahmed M.A., Shehata G.A., Eltahtawy M.A. (2009). Cognitive impairment after cerebrovascular stroke: Relationship to vascular risk factors. Neuropsychiatr. Dis. Treat..

[7] Douiri A., Rudd A.G., Wolfe C.D. (2013). Prevalence of poststroke cognitive impairment: South London stroke register 1995–2010. Stroke.

[8] Huang Y., Wang Q., Zou P., He G., Zeng Y., Yang J. (2023). Prevalence and factors influencing cognitive impairment among the older adult stroke survivors: A cross-sectional study. Front. Public Health.

[9] Vaishya R., Misra A., Vaish A., Ursino N., D’Ambrosi R. (2024). Hand grip strength as a proposed new vital sign of health: A narrative review of evidences. J. Health Popul. Nutr..

[10] Li G., Lu Y., Shao L., Wu L., Qiao Y., Ding Y., Ke C. (2023). Handgrip strength is associated with risks of new-onset stroke and heart disease: Results from 3 prospective cohorts. BMC Geriatr..

[11] Lopez-Jaramillo P., Lopez-Lopez J.P., Tole M.C., Cohen D.D. (2022). Muscular strength in risk factors for cardiovascular disease and mortality: A narrative review. Anatol. J. Cardiol..

[12] Roberts H.C., Syddall H.E., Sparkes J., Ritchie J., Butchart J., Kerr A., Cooper C., Sayer A.A. (2014). Grip strength and its determinants among older people in different healthcare settings. Age Ageing.

[13] Rijk J.M., Roos P.R., Deckx L., van den Akker M., Buntinx F. (2016). Prognostic value of handgrip strength in people aged 60 years and older: A systematic review and meta-analysis. Geriatr. Gerontol. Int..

[14] Alfaro-Acha A., Snih S.A., Raji M.A., Kuo Y.F., Markides K.S., Ottenbacher K.J. (2006). Handgrip strength and cognitive decline in older Mexican Americans. J. Gerontol. A Biol. Sci. Med. Sc..

[15] Boyle P.A., Buchman A.S., Wilson R.S., Leurgans S.E., Bennett D.A. (2010). Physical frailty is associated with incident mild cognitive impairment in community-based older persons. J. Am. Geriatr. Soc..

[16] Stessman J., Rottenberg Y., Fischer M., Hammerman-Rozenberg A., Jacobs J.M. (2017). Handgrip strength in old and very old adults: Mood, cognition, function, and mortality. J. Am. Geriatr. Soc..

[17] Veronese N., Stubbs B., Trevisan C., Bolzetta F., De Rui M., Solmi M., Sartori L., Musacchio E., Zambon S., Perissinotto E. (2016). What physical performance measures predict incident cognitive decline among intact older adults? A 4.4year follow up study. Exp. Gerontol..

[18] Turunen K.E., Laari S.P., Kauranen T.V., Uimonen J., Mustanoja S., Tatlisumak T., Poutiainen E. (2018). Domain-specific cognitive recovery after first-ever stroke: A 2-year follow-up. J. Int. Neuropsychol. Soc..

[19] Nys G., Van Zandvoort M., De Kort P., Jansen B., Van der Worp H., Kappelle L., De Haan E. (2005). Domain-specific cognitive recovery after first-ever stroke: A follow-up study of 111 cases. J. Int. Neuropsychol. Soc..

[20] Julious S.A. (2023). Sample Sizes for Clinical Trials.

[21] Chow S.C., Shao J., Wang H., Lokhnygina Y. (2017). Sample Size Calculations in Clinical Research.

[22] Machin D., Campbell M.J., Tan S.B., Tan S.H. (2011). Sample Size Tables for Clinical Studies.

[23] Zar J.H. (1999). Biostatistical Analysis.

[24] Nishiguchi S., Yamada M., Shirooka H., Nozaki Y., Fukutani N., Tashiro Y., Hirata H., Yamaguchi M., Tasaka S., Matsushita T. (2016). Sarcopenia as a risk factor for cognitive deterioration in community-dwelling older adults: A 1-year prospective study. J. Am. Med. Dir. Assoc..

[25] Chen L.K., Woo J., Assantachai P., Auyeung T.W., Chou M.Y., Iijima K., Jang H.C., Kang L., Kim M., Kim S. (2020). Asian Working Group for Sarcopenia: 2019 consensus update on sarcopenia diagnosis and treatment. J. Am. Med. Dir. Assoc..

[26] Kang Y., Na D., Hahn S. (2003). Seoul Neuropsychological Screening Battery.

[27] Julious S.A. (2005). Sample size of 12 per group rule of thumb for a pilot study. Pharm. Stat..

[28] Shaughnessy K.A., Hackney K.J., Clark B.C., Kraemer W.J., Terbizan D.J., Bailey R.R., McGrath R. (2020). A narrative review of handgrip strength and cognitive functioning: Bringing a new characteristic to muscle memory. J. Alzheimers Dis..

[29] Proust-Lima C., Amieva H., Dartigues J.F., Jacqmin-Gadda H. (2007). Sensitivity of four psychometric tests to measure cognitive changes in brain aging-population–based studies. Am. J. Epidemiol..

[30] Jaeger J. (2018). Digit symbol substitution test: The case for sensitivity over specificity in neuropsychological testing. J. Clin. Psychopharmacol..

[31] Chou M.Y., Nishita Y., Nakagawa T., Tange C., Tomida M., Shimokata H., Otsuka R., Chen L.K., Arai H. (2019). Role of gait speed and grip strength in predicting 10-year cognitive decline among community-dwelling older people. BMC Geriatr..

[32] Yang L., Koyanagi A., Smith L., Hu L., Colditz G.A., Toriola A.T., López Sánchez G.F., Vancampfort D., Hamer M., Stubbs B. (2018). Hand grip strength and cognitive function among elderly cancer survivors. PLoS ONE.

[33] Chen W.L., Peng T.C., Sun Y.S., Yang H.F., Liaw F.Y., Wu L.W., Chang Y.W., Kao T.W. (2015). Examining the association between quadriceps strength and cognitive performance in the elderly. Medicine.

[34] Rabin L.A., Paolillo E., Barr W.B. (2016). Stability in test-usage practices of clinical neuropsychologists in the United States and Canada over a 10-year period: A follow-up survey of INS and NAN members. Arch. Clin. Neuropsychol..

[35] Sachs A., Rising K., Beeson P.M. (2020). A retrospective study of long-term improvement on the Boston Naming test. Am. J. Speech Lang. Pathol..

[36] Ballard C., Rowan E., Stephens S., Kalaria R., Kenny R.A. (2003). Prospective follow-up study between 3 and 15 months after stroke: Improvements and decline in cognitive function among dementia-free stroke survivors >75 years of age. Stroke.

[37] Lo J.W., Crawford J.D., Desmond D.W., Bae H.J., Lim J.S., Godefroy O., Roussel M., Kang Y., Jahng S., Köhler S. (2022). Long-term cognitive decline after stroke: An individual participant data meta-analysis. Stroke.

[38] Ward N.S., Newton J.M., Swayne O.B., Lee L., Frackowiak R.S., Thompson A.J., Greenwood R.J., Rothwell J.C. (2007). The relationship between brain activity and peak grip force is modulated by corticospinal system integrity after subcortical stroke. Eur. J. Neurosci..

[39] Ismail S.S., Mohamad M., Syazarina S.O., Nafisah W.Y. (2014). Hand grips strength effect on motor function in human brain using fMRI: A pilot study. J. Phys. Conf. Ser..

[40] Heuninckx S., Wenderoth N., Debaere F., Peeters R., Swinnen S.P. (2005). Neural basis of aging: The penetration of cognition into action control. J. Neurosci..

[41] Olivier E., Davare M., Andres M., Fadiga L. (2007). Precision grasping in humans: From motor control to cognition. Curr. Opin. Neurobiol..

[42] Girgenti S.G., Brunson A.O., Marsh E.B. (2023). Baseline function and rehabilitation are as important as stroke severity as long-term predictors of cognitive performance post-stroke. Am. J. Phys. Med. Rehabil..

